# Genome assembly of the popular Korean soybean cultivar Hwangkeum

**DOI:** 10.1093/g3journal/jkab272

**Published:** 2021-07-30

**Authors:** Myung-Shin Kim, Taeyoung Lee, Jeonghun Baek, Ji Hong Kim, Changhoon Kim, Soon-Chun Jeong

**Affiliations:** 1 Bio-Evaluation Center, Korea Research Institute of Bioscience and Biotechnology, Cheongju, Chungbuk 28116, Republic of Korea; 2 Plant Immunity Research Center, Interdisciplinary Program in Agricultural Genomics, College of Agriculture and Life Sciences, Seoul National University, Seoul 08826, Republic of Korea; 3 Bioinformatics Institute, Macrogen Inc., Seoul 08511, Republic of Korea

**Keywords:** *Glycine max*, genetic map, genome assembly, soybean, structural variation

## Abstract

Massive resequencing efforts have been undertaken to catalog allelic variants in major crop species including soybean, but the scope of the information for genetic variation often depends on short sequence reads mapped to the extant reference genome. Additional *de novo* assembled genome sequences provide a unique opportunity to explore a dispensable genome fraction in the pan-genome of a species. Here, we report the *de novo* assembly and annotation of Hwangkeum, a popular soybean cultivar in Korea. The assembly was constructed using PromethION nanopore sequencing data and two genetic maps and was then error-corrected using Illumina short-reads and PacBio SMRT reads. The 933.12 Mb assembly was annotated as containing 79,870 transcripts for 58,550 genes using RNA-Seq data and the public soybean annotation set. Comparison of the Hwangkeum assembly with the Williams 82 soybean reference genome sequence (Wm82.a2.v1) revealed 1.8 million single-nucleotide polymorphisms, 0.5 million indels, and 25 thousand putative structural variants. However, there was no natural megabase-scale chromosomal rearrangement. Incidentally, by adding two novel subfamilies, we found that soybean contains four clearly separated subfamilies of centromeric satellite repeats. Analyses of satellite repeats and gene content suggested that the Hwangkeum assembly is a high-quality assembly. This was further supported by comparison of the marker arrangement of anthocyanin biosynthesis genes and of gene arrangement at the *Rsv*3 locus. Therefore, the results indicate that the *de novo* assembly of Hwangkeum is a valuable additional reference genome resource for characterizing traits for the improvement of this important crop species.

## Introduction

Hwangkeum is an important soybean [*Glycine max* (L.) Merr.] cultivar with distinctive organoleptic and agronomical features. Ever since its cultivar release in 1979 (Park *et al.* 1981), it has been widely grown and widely used as a breeding parent in Korea. According to the 2008 national survey report (Yu *et al.* 2008), it was used as a parent or grandparent in 19 of the 105 newly bred soybean cultivars released in Korea up to 2007. Hwangkeum has a determinate growth habit and nonshattering pods and is adapted to the middle Korean peninsula (Maturity Group V). Seeds are large (25 g per 100 seeds), round-shaped, and clear golden with yellow seed-coats and buff hila (Yang *et al.* 2010). Hwangkeum was found to be resistant to all soybean mosaic virus (SMV) strain groups identified in the USA (Chen *et al.* 2002), and the resistance was found to be conferred by multiple genes (Jeong and Jeong 2014). The genes controlling anthocyanin biosynthesis are highly polymorphic between Hwangkeum and IT182932, a wild soybean accession (Yang *et al.* 2010). Low isoflavone content in Hwangkeum led to the identification of novel loci that regulate the content of isoflavone (Yang *et al.* 2011).

The first genome sequence of soybean, one of the major seed crop species worldwide, was that of Williams 82, which was published in 2010 (Schmutz *et al.* 2010). The Williams 82 genome sequence was generated using a whole-genome shotgun approach with Sanger sequencing, and then assembled with physical and high-density genetic maps. Since then, this genome has been widely used as the reference genome for soybean research and has been updated up to version 4 (Song *et al.* 2016; Valliyodan *et al.* 2019). Additional genome assemblies that were supposed to represent soybean growing areas have been generated with high-throughput sequencing platforms: Japanese cultivar Enrei (Shimomura *et al.* 2015), Chinese cultivar Zhonghuang 13 (Shen *et al.* 2018), and southern US cultivar Lee (Valliyodan *et al.* 2019), while classifying Williams 82 as a northern US cultivar. In addition, the genome sequences of two wild soybean accessions W05 (Xie *et al.* 2019) and PI 483463 (Valliyodan *et al.* 2019), and of a perennial relative of soybean, *Glycine latifolia* (Liu *et al.* 2018), have already been published. These efforts have recently culminated in the construction of a high-quality pan-genome from 26 diverse soybean accessions sequenced individually using single molecule real-time (SMRT) sequencing, together with the existing Williams 82, Zhonghuang 13, and W05 genomes (Liu *et al.* 2020).

Degrees of structural variation of these genome sequences from that of Williams 82 are highly variable. For example, comparison between those of Williams 82 and Zhonghuang 13 revealed many putative mega-scale SVs, while none were observed between those of Williams 82 and Lee. Here, we report our investigation of the Hwangkeum genome using PromethION nanopore sequencing data and two genetic maps. We show that most of the mega-scale SVs between Hwangkeum and Williams 82 assemblies might be assembly errors. Besides those mega-scale variations, most of the small and SVs between the two genome assemblies might be natural. The observed differences were validated by examination of known variation regions, including anthocyanin biosynthesis genes and disease resistance genes.

## Materials and methods

### Plant materials and sequencing

Seeds of Hwangkeum whose breeding line was known as Suwon 97 (Chen *et al.* 2002; Jeong and Jeong 2014) were planted in the greenhouse at the Korea Research Institute of Bioscience and Biotechnology. After three weeks’ growth, a bulk of young trifoliolate leaf tissues was collected for genomic DNA extraction. Note that the seeds of Hwangkeum used in this study came from the line of Hwangkeum that had been subject to single plant selection at least twice during our recent 180K SoyaSNP array and genome resequencing studies (Lee *et al.* 2015; Kim *et al.* 2021). Genomic DNAs for the generation of Illumina short-read (Illumina, San Diego, CA, USA) and PacBio SMRT long-read sequences (Pacific Biosciences, Menlo Park, CA, USA) were extracted using the CTAB method, as described by Saghai-Maroof *et al.* (1984). Paired-end and mate-pair libraries for Illumina short-read sequencing were prepared, and then sequenced using a HiSeq 2500 System. A library for PacBio SMRT sequencing was prepared using SMRTbell Express Templates with Sequel SMRT Cell 1M v2, Sequel Binding Kit 2.1, and was then sequenced with a PacBio Sequel system. Genomic DNA for the single-molecule sequencer PromethION (Oxford Nanopore Technologies Ltd., Oxford, UK) sequencing was extracted using Nanobind Plant Nuclei Big DNA Kit—Alpha Version (#NB-900-801-01) (Circulomics Inc., Baltimore, MD, USA), as described by Workman *et al.* (2018), and was further purified using 26G Needle shearing and Bluepippin size selection [High Pass Plus (20–150) kb]. The purified DNA was then prepared for sequencing following the protocol in the genomic sequencing kit SQK-LSK109 (Oxford Nanopore Technologies Ltd.).

For the extraction of total RNAs, plants were further grown to a pod-bearing stage, and the bulked tissues were separately collected. Total RNAs were extracted from the six different tissues using RNeasy Plant Mini Kit, following the manufacturer’s instructions (QIAGEN, Venlo, the Netherlands). Two separately combined RNA extracts were used for RNA sequencing (RNA-seq). Equal amounts of the RNA extracted from immature seeds, young shoot, and young stems were combined into one sample, and the RNA from flowers, leaves, and roots were combined to form another sample. Libraries for each of the RNA samples were prepared using TruSeq RNA Sample Prep Kit v2 (Illumina), and then 101 bp paired-end short reads were generated on an Illumina platform.

### Genome assembly

#### PacBio SMRT data

Assembly of SMRT subreads was performed with FALCON-Unzip to produce primary contigs (Chin *et al.* 2016). The primary contigs were polished with mapped PacBio subreads with Quiver implementation in variantCaller tool (SMRT Link 6.0.0.47841; https://www.pacb.com/support/software-downloads/) with three iterations, followed by Pilon (v1.22) (Walker *et al.* 2014) with Illumina data. Mate-pair reads were used to construct scaffolds with the SSPACE program (v2.3.1) (Boetzer *et al.* 2011), with sequence gaps filled with PBJelly (v15.8.24) (English *et al.* 2014). The scaffolding and gap-filling were then repeated with paired-end reads. Finally, ALLMAPS (Tang *et al.* 2015) was used to construct the 20 pseudo-chromosomes by anchoring the assembled contigs/scaffolds to two genetic maps (WH and HI maps) that had been constructed using Hwangkeum as a parental line (Lee *et al.* 2020). In our previous study, we constructed four high-density genetic maps from Williams 82K (*G. max*) by Hwangkeum (*G. max*) (referred to as WH), Hwangkeum by IT182932 (*Glycine soja*) (HI), Williams 82K by IT182932 (WI), and IT182932 by IT182819 (*G. soja*) (II) populations. To remove missing markers in the assemblies, probe or primer sequences of markers were searched against the assembly using BLAST+ (Camacho *et al.* 2009), and the marker sequences hit by >95% identity and >88% coverage were input into the ALLMAPS program, with equal weight assigned to the two genetic maps.

#### Nanopore PromethION data

All PromethION reads were assembled into contigs with Shasta v.0.1.0 (Shafin *et al.* 2020) to obtain raw genome assembly results. Then, ALLMAPS (Tang *et al.* 2015) was used to construct the 20 pseudo-chromosomes, as described above. The resulting assemblies were polished with Pilon (v1.22) (Walker *et al.* 2014) with three iterations with mapping of Illumina short reads, and with Arrow implemented SMRT Link 8.0.0.80529 with three iterations with mapping of SMRT reads. To assess the completeness of the final genome, Benchmarking Universal Single-Copy Orthologs (BUSCO) (Simão *et al.* 2015) was employed using eukaryota odb10 (creation date: November 20, 2019, number of species: 70, number of BUSCOs: 255) and embryophyta odb10 (creation date: November 20, 2019, number of species: 50, number of BUSCOs: 1614) core conserved genes as databases.

### Comparative genomics between Williams 82 and Hwangkeum

We identified SNPs and indels (<50 bp) using paftools.js from the minimap2 distribution (Li 2018). Briefly, we mapped the Hwangkeum assembly as a query against the Williams 82 (Wm82.a2.v1) assembly as a reference using minimap2, and called variants through the paftools.js module in minimap2 with the following flags (minimap2 -c –cs ref.fasta query.fasta | sort -k6,6 -k8,8n | paftools.js call -L15000). In this study, we decided to use the Williams 82 version 2 assembly (Wm82.a2.v1) for comparative genomics between Williams 82 and Hwangkeum. Hereafter, unless otherwise specified, the version of the Williams 82 assembly is Wm82.a2.v1. The version 3 and 4 of the Williams 82 assembly have been recently reported that incorporated additional targeted sequencing to fill gaps in the version 2 (Valliyodan *et al.* 2019). However, full details of the version 3 and 4 have yet to be published. We identified and classified the SVs using the SVs from MUMmer (SVMU) pipeline (Chakraborty *et al.* 2018; Marçais *et al.* 2018). Insertion (INS) or deletion (DEL) was classified on the basis of whether the Hwangkeum assembly had longer or shorter sequence, respectively, with respect to the reference genome Williams 82 sequence. Translocation and inversion events (both refer to structure variation ≥1.0 Kbp) for the Hwangkeum assembly were detected by their locations and orientations to their neighboring blocks relative to the reference genome using MUMmer4 (v. 4.0.0beta2) (Marçais *et al.* 2018), and were then substantiated by manually checking collinearity between the physical and genetic locations of markers available on scaffolds and genetic maps.

Visual evaluations for structural comparisons between assemblies were made using dot plots generated by the MUMMERPLOT utility from MUMMER v.4.0 (Marçais *et al.* 2018). Correspondences of orthologous genes between Hwangkeum and Williams 82 were determined using OrthoMCL (v2.0.9) with default options (Li *et al.* 2003). We used the MCscan (Python version) (Tang *et al.* 2008) to compare gene arrangement at the *Rsv*3 locus between the Hwangkeum and Williams 82 assemblies.

### Analysis of telomeric and centromeric repeats

As a measure of pseudomolecule completeness near the chromosome ends, we checked for characteristic telomeric repeat motifs AAACCCT and AGGGTTT within 1500 bases of the leading and trailing ends of the pseudomolecule ends (Valliyodan *et al.* 2019). In addition, we searched for any novel repeat elements in the terminal sequences with Tandem Repeats Finder (Benson 1999).

We searched for two centromere-specific satellite repeats (CentGm-1 and CentGm-2), which have been predicted using sequencing data (Vahedian *et al.* 1995; Swaminathan *et al.* 2007; Gill *et al.* 2009; Tek *et al.* 2010), and then confirmed experimentally (Gill *et al.* 2009; Findley *et al.* 2010), in order to identify the assembled centromeric regions in the Hwangkeum and Williams 82 assemblies. Representative consensus sequences of CentGm-1 and -2 were proposed from the analysis of three soybean assemblies by Valliyodan *et al.* (2019). These representative satellite repeat consensus sequences were aligned with the Williams 82 and Hwangkeum assemblies with an -evalue 1e-5 -task blastn-short -penalty -1 option in BLASTN to estimate the location and length of the centromeres on the pseudomolecules. All the repeat sequences hit by each of the CentGm-1 and -2 sequences had >67% sequence identity with their query sequences. We then further filtered these candidate repeats with <80% alignment coverage. Note that <80% alignment coverage and <60% sequence identity were cut-off criteria used in a previous phylogenetic analysis of a whole-genome shotgun database (Gill *et al.* 2009). A majority of repeat sequences hit by each of the CentGm-1 and -2 sequences appeared to overlap each other, likely due to the 81.5% sequence identity between the CentGm-1 and CentGm-2, and thus the two extracted sequence sets for each of the Hwangkeum and Williams 82 assemblies were combined into a set of repeat sequences by removing one of the overlapped sequences. Lengths of the satellite tandem repeats in pseudomolecules and unanchored contigs were determined with the Tandem Repeat Finder (Benson 1999).

The combined repeats from the Hwangkeum assembly were further filtered for efficient phylogenetic analysis. First, 4599 repeats with length <89 bp and 38 with >94 bp were excluded. The cd-hit-est software was then used to cluster similar repeat sequences into clusters using the parameters “-c 0.90 -n 10” within a set of 20,386 satellite repeats (Fu *et al.* 2012). Multiple sequence alignment of the resultant nonredundant 4469 satellite repeats was performed with ClustalW (Larkin *et al.* 2007), and then phylogenetic analysis of the aligned sequences was performed with MEGA7 software using the neighbor-joining method (Kumar *et al.* 2016). In this phylogenetic analysis, four CentGm-1 (referred to as CentCm-1_AF, CentCm-1_E, CentGm-1_Gill, and CentCm-1_J2), three CentGm-2 (CentCm-2_G, CentCm-2_Gill, and CentCm-2_M) representative sequences used for karyotyping soybean by Findley *et al.* (2010), and two (CentGm-1_V and CentGm-2_V) consensus sequences proposed by Valliyodan *et al.* (2019) were included as reference sequences to infer the already established CentGm-1 and CentGm-2 repeat subfamilies.

### Genome annotation

Repetitive sequences were identified with RepeatMasker (v. 4.1.1; http://repeatmasker.org) with -s -pa 15 -no_is -xsmall -gff -lib options using a soybean repeat library from SoyTEdb (Du *et al.* 2010). We annotated gene models using the Seqping pipeline (Chan *et al.* 2017) with slight modifications. Seqping uses transcriptome data and three self-training Hidden Markov Model (HMM) models, and the resultant predictions are then combined using MAKER2 (Holt and Yandell 2011). We added protein models at the MAKER2 step. The predicted genes were filtered out using e-AED value with threshold of 0.4. For the transcript data to train the prediction models, we used RNA-seq data generated from the Hwangkeum tissues described above. The RNA-seq data were processed with genome-guide assembly, and gene structures were then predicted by the EMBOSS getorf program with the default parameters. All the resultant gene model sets were integrated into single RNA-seq-based gene model sets. Soybean protein set that was searched using keywords of “*G.* *max*”[porgn: _txid3847] or “Glycine soja”[porgn : _txid3848] in the NCBI protein database (accessed on May 22, 2020) was used as a reference protein file for the validation and annotation of the gene predictions. We used tRNAscan-SE software (version 2.0) with default parameters for tRNA annotation (Chan and Lowe 2019) and Barrnap 0.9 (https://github.com/tseemann/barrnap) for rRNA annotation. Protein function annotations were added by searching for homologous proteins in the UniProt SwissProt database (accessed on June 1, 2018) (Bateman *et al.* 2017) using BLASTP and eggNOG v4.5 database (Huerta-Cepas *et al.* 2016) using psi-blast with *E*-value < 1e-5, num_alignments 5, and num_descriptions 5, and protein domains using InterProScan 5.34-73.0 (Finn *et al.* 2017). The functional annotation results were read using Annie (http://genomeannotation.github.io/annie/), and then genome annotation summary statistics were generated using the software GAG (Geib *et al.* 2018).

Nucleotide-binding and leucine-rich-repeat (NLR) genes, which are members of the largest resistance gene family in plants, were predicted using TGFam-Finder (v. 1.03) (Kim *et al.* 2020). TGFam-Finder is a domain search-based gene annotation tool. We used the NB-ARC domain (PfamID = PF00931) (van der Biezen and Jones 1998), which was used in the TGFam-Finder program, as TARGET_DOMAIN_ID for searching NLR genes. Transcriptome mapping was performed using the RNA-seq data generated from the Hwangkeum tissues described above. We searched for only primary transcripts from the Hwangkeum genome sequence.

## Results and discussion

### Genome assembly of the Hwangkeum

The genome of *G.* *max* cv. Hwangkeum was sequenced at 78× coverage (78,861,723,603 bases) using PacBio SMRT technology, and at 89× coverage (89,519,105,740 bases) using Nanopore PromethION technology. Both the sequencing data were separately assembled with error corrections up to pseudomolecules. The diploid FALCON-Unzip assembler produced an initial SMRT-based contig assembly with 1436 primary contigs, N50 of 1.71 Mb, and a total length of 963.13 Mb (Supplementary Table S1). After error corrections and scaffolding using Illumina mate-pair and paired-end reads, the final primary assembly was scaffolded into 730 scaffolds covering 966.25 Mb with an N50 of 2.54 Mb and with a maximum length of 11.72 Mb (Supplementary Table S2). We initially evaluated two recently published assemblers, Shasta and wtdbg2 (Ruan and Li 2020; Shafin *et al.* 2020), on our PromethION read data (Supplementary Table S1). Total lengths of both assemblies from the PromethION data were approximately 30 Mb shorter than that from the SMRT data. The Shasta assembly showed approximately 8 times fewer number of contigs (847) and 10 times longer N50 length (6.95 Mb) relative to those of the wtdbg2 assembly. Thus, the results showed that, despite much higher levels of differences, the tendency was somewhat consistent with that from the human genome assembly study (Shafin *et al.* 2020), suggesting that Shasta might be more appropriate than wtdbg2 for the assembly of our Hwangkeum PromethION sequencing data.

To further evaluate which of the FALCON-Unzip SMRT and Shasta PromethION assemblies was superior, we then generated chromosome-scale pseudomolecules by ordering and orienting the assembled contigs/scaffolds via anchoring to two genetic maps that had been constructed using Hwangkeum as a parental line (Lee *et al.* 2020). Our comparison between four genetic maps, including the two Hwangkeum genetic maps, showed excellent collinearity with no marker order difference, although there appeared to be putative megabase-scale inversions based on the lack of cross-overs. Thus, we hypothesized that the assembly that showed the lesser number of discrepant markers between sequence assembly and genetic maps was likely superior to the other. The final assembly of Hwangkeum on the SMRT data consisted of 944.02 Mb of 20 chromosome-level pseudomolecules containing 640 scaffolds and 22.32 Mb of 90 unplaced scaffolds, while that on the PromethION data consisted of 907.90 Mb of 20 chromosome-level pseudomolecules containing 399 contigs and 19.74 Mb of 448 unplaced contigs. Thus, approximately 30 Mb longer sequences of SMRT scaffolds relative to that of the PromethION contigs were anchored to 20 chromosome-scale pseudomolecules. For the SMRT pseudomolecules, 553.39 Mb of 201 scaffolds were oriented with genetic markers, while 634.65 Mb of 90 contigs for the PromethION pseudomolecules were oriented (Supplementary Table S3), suggesting that the approximately 80 Mb sequence was better oriented in the PromethION assembly than in the SMRT assembly. We then examined the number of translocation errors, which represent breaks in collinearity between sequence and genetic maps markers due to the mixing of nonhomologous chromosomes as well as of the assembled pseudomolecules, in order to assess the integrity of scaffolds or contigs. From the SMRT pseudomolecule assembly, we observed 45 single-marker inter-chromosomal translocation errors, 121 multiple marker chimeric scaffolds with mappings to multiple linkage groups, and one apparent intra-chromosomal translocation on chromosome 13. In stark contrast, we observed only one chimeric scaffold on chromosome 18 from the PromethION pseudomolecule assembly. Three markers at the top of chromosome 18 appeared to best match with three different regions on chromosome 11. The results indicated that the PromethION-based assembly contained a much lower number of errors than the SMRT-based assembly in this study. Thus, we decided to use the PromethION-based assembly as a representative assembly of Hwangkeum genome in this study.

The initial PromethION-based assembly was then error-corrected using Pilon with the Illumina short reads and Arrow with the SMRT reads, which was a similar strategy to those used in the other plant genome assemblies (Xie *et al.* 2019; Jiao and Schneeberger 2020). When we mapped marker sequences from the WH and HI maps to the error-corrected assembly, we observed that the three markers at the top of chromosome 18 that best matched with the three different regions on chromosome 11 in the initial ALLMAPS assembly now best matched with the top region of chromosome 18. The final error-corrected Nanopore PromethION assembly had a total length of 933.12 Mb, and consisted of 913.20 Mb of 20 chromosome-level pseudomolecules containing 378 contigs and 19.92 Mb of 448 unplaced contigs (Table 1).

**Table 1:** Summary statistics of the Hwangkeum genome assembly

Assembly feature	Number	Size
Total assembly length		933,123,489 bp
Pseudomolecules	20	913,200,796 bp
Unanchored contigs	448	19,922,693 bp
Repetitive content		468,186,948 bp (50.17%)
Centromeric satellite repeats	25,030	2,249,110 bp (0.24%)
Number of transcripts	79,870	
Number of genes	58,550	

### Evaluation of the assembly genome quality

Analyses with two BUSCO databases, eukaryota odb10 and embryophyta odb10, indicated that the genome content was effectively captured in the Nanopore PromethION assembly (Supplementary Table S4): BUSCO analysis against eukaryota odb10 and embryophyta odb10 demonstrated 2/255 (0.7%) and 15/1614 (0.9%) of BUSCO genes missing from the assembly, respectively. We found telomeric repeat motifs AAACCCT and AGGGTTT on only 9 of the 40 pseudomolecule ends in Hwangkeum relative to 23 in the Williams 82 reference sequence. The results indicated that although our PromethION sequencing is not nearly as efficient as Sanger shotgun sequencing, it caught the ends of chromosomes.

We also evaluated distribution patterns of centromeric satellite repeats across chromosomes in the Hwangkeum assembly. Two subfamilies of centromere-specific satellite repeat sequences (CentGm-1 and CentGm-2 with 92-bp and 91-bp monomers, respectively) have been reported using sequencing data (Vahedian *et al.* 1995; Swaminathan *et al.* 2007; Gill *et al.* 2009; Tek *et al.* 2010), and then confirmed by immunoprecipitation (Tek *et al.* 2010) and fluorescent *in situ* hybridization (Gill *et al.* 2009; Findley *et al.* 2010). Representative consensus sequences of CentGm-1 and -2 were recently proposed from the analysis of three soybean assemblies (Valliyodan *et al.* 2019), and thus we used these two sequences to identify the assembled centromeric regions in the Hwangkeum and Williams 82 assemblies. After filtration with a cutoff criterion of <80% alignment coverage, we obtained 24,066 CentGm-1 and 22,046 CentGm-2 repeat sequences from the Hwangkeum assembly and 96,563 CentGm-1 and 92,749 CentGm-2 repeat sequences from the Williams 82 assembly. Thus, our cutoff threshold was less stringent than that used by Valliyodan *et al.* (2019) because they extracted only 11,829 CentGm repeats from the Williams 82 assembly. As expected from the 81.5% sequence identity between CentGm-1 and CentGm-2, a total of 21,612 repeat sequences were hit by both the query repeat sequences and thus their locations overlapped each other. Thus, the two extracted sequence sets from the Hwangkeum assembly were combined into a set of 25,030 repeat sequences (∼2.3 Mbp) (Table 1). Of the 25,030, the positions of 23,494 (93.8%) appeared to be head-to-tail tandem repeats, a feature typical of centromeric satellite repeats (Jiang *et al.* 2003). When their number, size, and locations were verified using Tandem Repeat Finder (Benson 1999), 24,859 (99.3%) of them appeared to be direct head-to-tail tandem repeats (Supplementary Table S5 and File S1). The 91-bp CentGm-2 repeats were nearly absent (<20 copies) on chromosome 18 and the 92-bp CentGm-1 repeats were absent on chromosomes 1 and 7 and nearly absent (<20) on chromosomes 6, 9, 10, and 11. Thus, our results are somewhat consistent with a previous observation (Valliyodan *et al.* 2019) that copy numbers of identified tandem repeat units were highly variable between chromosomes, although this study showed wider distribution of the 91-bp CentGm-2 repeats across chromosomes unlike the previous observation. In the case of the Williams 82 assembly, we obtained a final combined set of 100,654 repeat sequences (∼9.2 Mb). Of the 100,654 repeats, 93,456 (92.8%) appeared to be head-to-tail tandem repeats. About 40.8% of the repeat sequences in the Hwangkeum assembly and ∼51.3% of them in the Williams 82 reference assembly were located in unanchored scaffolds, indicating that almost half of the highly repeated centromeric repeats were not incorporated into pseudomolecules. Our observation that the total numbers of centromeric repeats were approximately four times higher in the Williams 82 reference assembly than in the Hwangkeum assembly suggests that the assembly collapse of centromeric repeats due to length and nucleotide errors prevalent in nanopore basecalling is likely a main cause of the difference of total lengths of assemblies between Williams 82 and Hwangkeum (Tørresen *et al.* 2019; Shafin *et al.* 2020).

### Genome structure comparison between Hwangkeum and Williams 82 genomes

Our recent genetic map study showed multiple mega-scale discordant regions between the Williams 82 reference genome and our genetic maps (Lee *et al.* 2020). However, comparison between the Williams 82 and Lee genome sequences resulted in no mega-scale SV (Valliyodan *et al.* 2019). In contrast, comparison between the Williams 82 and Zhonghuang 13 genome sequences identified many large (>100 kb) structural variants (SV), including four mega-scale SVs (Shen *et al.* 2018, 2019). However, detailed investigations of whether the mega-scale SVs are real or miss-assemblies in either the assembly were not reported; neither did their subsequent pan-genome study address these mega-scale SVs (Liu *et al.* 2020). Thus, rather than comparing our Hwangkeum genome sequence and all other soybean *de novo* assemblies available, we decided in this study to focus on comparison between the current Hwangkeum and the Williams 82 reference genome.

Direct comparison between corresponding chromosome sequences of the Hwangkeum and Williams 82 assemblies identified 1,788,320 SNPs and 517,907 indels (<50 bp) (Supplementary Table S6 and File S2). The number of SNPs was similar to that observed between the genome assemblies of Lee and Williams 82 (Valliyodan *et al.* 2019) and the numbers of both SNPs and indels were similar to those between ZH13 and its closer accessions of 26 *de novo* assembled genomes, thereby suggesting a substantial distance between Hwangkeum and Williams 82 genomes (Liu *et al.* 2020). However, several chromosomal regions showed no difference between the Hwangkeum and Williams 82 assemblies. For example, the 85-cM gap in the middle of chromosome 4 for the WH population detected in our previous genetic mapping study (Lee *et al.* 2020) contained 15 no-variation regions of >200 kb with the largest one of 1.72 Mb. These appear to be identity-by-descent regions inherited from a common ancestor during soybean breeding history.

**Figure 1:**
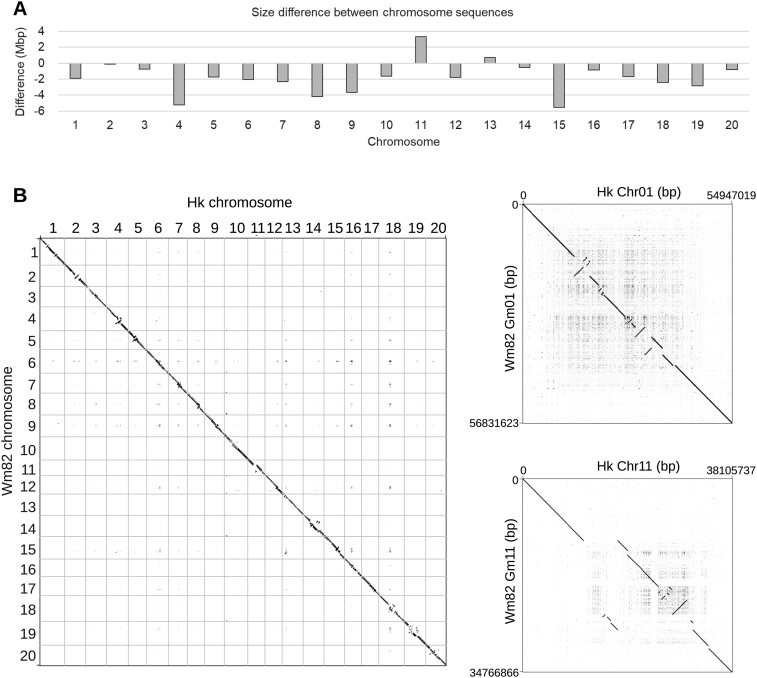
Comparison between the Hwangkeum and Williams 82 assemblies. (A) Bar chart that shows size difference values between corresponding chromosomes of the Hwangkeum and Williams 82 assemblies. The values were obtained by subtracting length of each chromosome in the Williams 82 assembly from that of the corresponding chromosome in the Hwangkeum assembly. (B) Dot plots showing alignments of 20 chromosome sequences between the Hwangkeum (Hk) assembly and Williams 82 (Wm82) reference genome assembly and showing alignments of individual chromosomes 1 and 11 between the Hk and Wm82 assemblies.

In addition to the difference in the number and locations of centromeric repeats between the Hwangkeum and Williams 82 assemblies, most of the chromosomes in the Hwangkeum assembly were shorter in size, with a median decrease of 1.76 Mb, relative to corresponding chromosomes in the Williams 82 assembly (Figure 1A). Notable outliers were two of the greatest decreases that occurred in chromosomes 4 and 15, and increases observed in chromosomes 11 and 13. Aligning the Hwangkeum assembly to the Williams 82 assembly, we found additional notable megabase-scale rearrangements in these exceptionally decreased or increased chromosomes as well as in the other chromosomes (Figure 1B and Supplementary Figure S1). All those mega-scale rearrangements located at the presumed pericentromeric regions where genetic markers are not resolved well due to low recombination rate. Interestingly, those exceptionally decreased or increased chromosomes could be explained by the insertions of unanchored scaffolds present in the Williams 82 assembly (chromosome 11) or by the corrected positioning of misjoints predicted by our genetic mapping study (chromosomes 4, 13, and 15), as described below.

**Figure 2:**
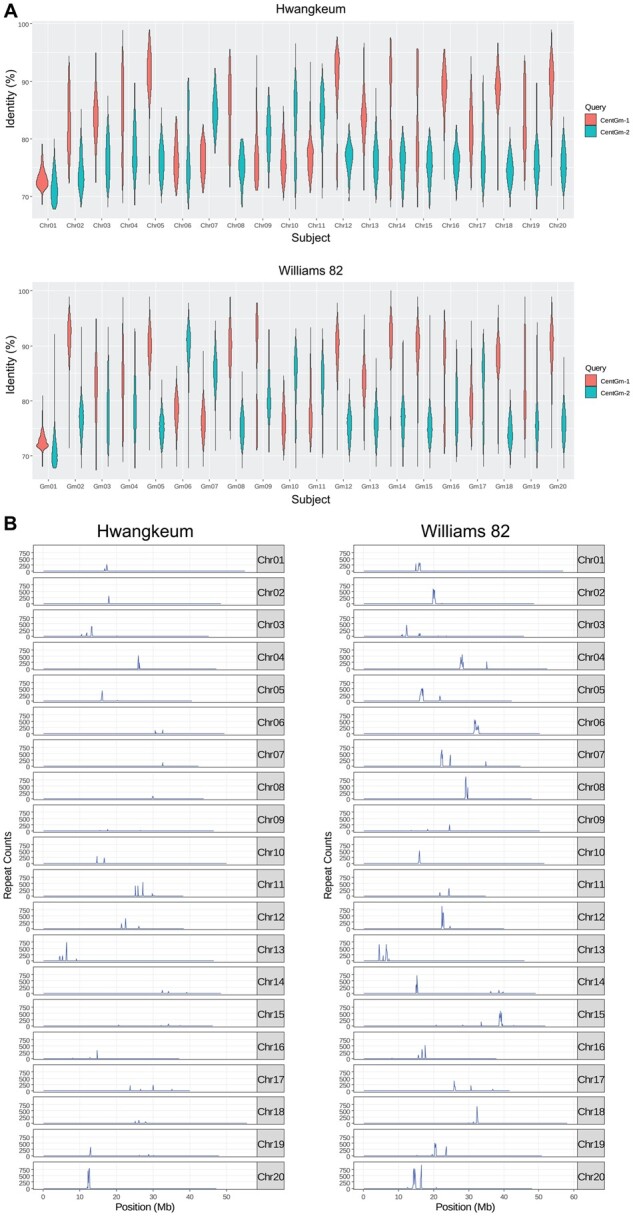
Genome-wide distribution patterns of centromeric repeats in the Williams 82 and Hwangkeum assemblies. (A) Violin plot distributions of the percent identity of centromeric repeats hit by BLAST searches with CentGm-1 and CentGm-2, respectively, along the 20 soybean chromosomes, as sampled in the Hwangkeum and Williams 82 assemblies. (B) Genome-wide centromeric repeat density in the Hwangkeum and Williams 82 assemblies. Centromeric repeats hit by CentGm-1 or CentGm-2 were combined by removing one of overlapping repeat sequences and then the repeat sequence density was plotted in 100-kb windows along the 20 soybean chromosomes.

Searches of SVs in the Hwangkeum assembly relative to the Williams 82 reference sequence resulted in 11,542 deletions (≥50 bp), 10,845 insertions (≥50 bp), 2504 interchromosomal translocations (>1000 bp), and 168 inversions (>10 kbp) (Supplementary File S3). The total length of insertions (27.5 Mb) was 5.6 Mb longer than that of deletions (21.9 Mb). Our close examination suggested that the length difference was largely due to the insertions of unanchored scaffolds in the Williams 82 assembly. For example, most of scaffold_21 (3.57 Mb) and half of scaffold_22 (1.24 Mb), which are the two longest unanchored scaffolds in the Williams 82 assembly, were inserted with inverted orientation into chromosome 11. Scaffold_21 corresponded with the largest insertion of 3.46 Mb, and scaffold_22 corresponded with a cluster of several large (>7 kb) insertions that were likely separated by repetitive sequences. Therefore, the insertion of scaffold_21 and scaffold_22, which was also predicted by our previous genetic mapping study (Lee *et al.* 2020), is the main cause of the size increase of chromosome 11 in Hwangkeum relative to the Williams 82 reference sequence. Thus, this is not actual structural variation between Hwangkeum and Williams 82, but rather an improvement in the Hwangkeum assembly since scaffold_21 and _22 were not anchored in the Williams 82 assembly.

The sizes of the detected interchromosomal translocations ranged from 1001 to 184,994 bp with median of 3294 bp. When we searched for 109 putative misjoint chromosomal regions in the soybean Williams 82 reference genome sequence (Wm82.a2.v1), which required re-positioning to different chromosomes based on genetic maps constructed in our previous study (Lee *et al.* 2020), more than 80 regions were located at different chromosomes in the Hwangkeum genome, as predicted. The results demonstrate the soundness of our misjoint detection method, as well as the improvement in the Hwangkeum assembly. Those misjoint regions that required re-positioning by multiple markers tended to contain multiple adjacent blocks, and thus the adjacent blocks could be merged together to treat them as the same large misjoint event, in accordance with a previous method for human genome study (Audano *et al.* 2019). As expected, each of the merged blocks tended to correspond with a large indel longer than 100 kbp, thereby indicating evidence of another improvement in the Hwangkeum assembly. One exception is the movement of a 2.43 Mb fragment between the 36.99 and 39.42 Mb positions from chromosome 15 in Williams 82 to chromosomes 4 (approximately 0.38 Mb), 5 (0.57 Mb), and 13 (1.48 Mb) in the Hwangkeum (Supplementary File S3). Although no markers were located on these chromosomal regions in the WH and HI maps, the fragment in the Williams 82 assembly is likely a concatenated scaffold. Interestingly, these putative artifacts explained the relatively larger decrease of chromosome size in chromosome 15 and slight increase in chromosome 13. Despite the gain of the ∼0.38 Mb fragment, an approximately 0.83 Mb fragment was translocated from chromosome 4 (Williams 82) to chromosome 3 (Hwangkeum), as predicted by the genetic mapping, thereby partly explaining the decrease in the length of chromosome 4. Taken together, our results suggest that the difference of the total lengths of insertions and deletions is not the main cause for the shorter total assembly length of the Hwangkeum assembly than that of the Williams 82 assembly.

The detected 168 inversions comprised 64 inversions and 104 intrachromosomal translocation & inversions (Supplementary File S3). Among the predicted inversions, each of the 94 inversion fragments clearly matched with a single contig. Closer inspection of these inversion fragments indicated that because most of these contigs contained a single marker or multiple cosegregating markers in our WH and HI genetic maps, they could not be oriented in the ALLMAPS assembly process. Approximately 40 inversions that were part of a contig or covered by part of two contigs were located at low-recombination chromosomal regions, and so neither could their orientations be determined by genetic markers. At least 13 inversions were apparent errors by the ALLMAPS assembly because their orientations were inversed against the orders of the markers with one or two recombination events in the two genetic maps. All these putative artificial inversions were marked in the list of detected inversions (Supplementary File S3). Which of the Hwangkeum or Williams 82 assemblies, both of which used genetic maps for pseudomolecule construction, contains correct orientations for these putative artificial inversions is unknown at this point because most of them locate at low-recombination chromosomal regions. Excluding all these putative assembly errors, 27 predicted inversions remained to be real. In the results, most of the detected inversions were not supported by genetic markers, and only 27 detected inversions appeared to be imbedded within a contig and the total length of the inversions was 1.86 Mb. Among the 27, seven were supported by genetic marker orders. The sizes of the 27 inversions ranged from 10 to 211 kb. As the genome-wide average recombination rate in soybean was estimated to be 2.5 cM/Mb (Lee *et al.* 2013), this result suggests that the inversions may not have a substantial impact on the genetic difference between Hwangkeum and Williams 82. The two largest detected inversions were adjacent but not overlapping 211 and 201-kb fragments between 31.89 and 32.47 Mb positions on chromosome 7 in the Hwangkeum assembly. Although many of the breakpoint junctions of the detected inversions appeared to be located on repetitive sequences, we attempted to validate the two largest inversions by PCR-amplification using primers spanning their breakpoint junctions (Supplementary Figure S2). Sequence comparison between the Hwangkeum and Williams 82 assemblies suggested that there might be some possibility generating specific primers from one side of the 211-kb inversion and from both sides of the 201-kb inversion. However, only one primer set, which was designed for amplification of one breakpoint junction of the 201-kb inversion, gave a specific PCR product that was subsequently confirmed by sequencing, supporting the correct assembly of the Hwangkeum genome.

### Diversity and evolution of centromeric satellite repeats

The differences of the locations, numbers, and ratios of the two repeats that distributed across soybean chromosomes supported the notion that differential distributions of these distinct repeats may reflect the allopolyploid nature of soybean (Gill *et al.* 2009), and then were used for the karyotyping of 20 soybean chromosome pairs (Findley *et al.* 2010). As we identified nearly nine times more satellite repeats from the Williams 82 assembly, we decided to further investigate the distribution patterns and evolution of centromeric repeats across chromosomes to investigate the integrity of the Hwangkeum genome assembly. We first compared the two groups of satellite repeats hit by BLAST searches with CentGm-1 and CentGm-2, respectively, from the Hwangkeum and Williams 82 assemblies (Figure 2A and Supplementary Figure S3). The distribution patterns of percent identity values from the BLAST searches within each of the chromosomes could be divided into three groups. First, both CentGm-1- and CentGm-2-hit repeats showed lower than 80% identity (chromosomes 1, 6, 9, and 19), thereby suggesting that members of this first group might form novel subfamilies of soybean satellite repeats diverged from both the known CentGm-1 and CentGm-2 repeats (Gill *et al.* 2009). Particularly, all four categories of the hit repeats for chromosome 1 had lower than 73% median identity values. Second, the CentGm-1-hit repeats showed higher percent identity than the CentGm-2-hit repeats (chromosomes 2, 4, 3, 5, 8, 12, 13, 14, 15, 16, 17, 18, and 20). Third, the CentGm-1-hit repeats showed lower percent identity than the CentGm-2-hit repeats (chromosomes 7, 10, and 11). The distribution patterns could also be divided into two groups of narrow or wide identity value distributions. Despite the large difference of the numbers of repeats identified, the distribution patterns were quite similar between the Williams 82 and Hwangkeum assemblies. The results suggested that the higher diversity of repeat sequences within a chromosome might not be due to assembly errors but reflect polymorphisms of repeats generated during the evolution of each chromosome. Interestingly, approximately half of the unanchored contigs that are assumed to be subject to much less degree of assembly errors showed wide identity value distributions (Supplementary Figure S3).

The genomic distribution of the unique satellite repeats in 100-kb windows along the 20 soybean chromosomes showed that the centromere on each chromosome revealed different patterns of repeat density peaks (Figure 2B). Although the highest peaks of centromeric repeats between the two assemblies on most of the pseudomolecules corresponded to each other, the Williams 82 assembly showed more additional peaks. Notably, while the Williams 82 assembly showed two centromeric locations separated by more than 10 Mb from each other on chromosomes 7 and 14, Hwangkeum showed single locations on both the chromosomes. Five chromosomes (3, 4, 15, 19, and 20) in the Williams 82 assembly showed two centromeric locations separated by several Mb from each other. Separations of putative centromeric regions by more than 10 Mb were also observed on four chromosomes in the updated Zhonghuang 13 assembly (Shen *et al.* 2019). With some exceptions such as the point centromeres or holocentromeres, monocentric centromeres from plant to animal species are normally established on highly repetitive DNA arrays that usually contain distinct centromeric repeats (Cuacos *et al.* 2015; Barra and Fachinetti 2018). A fluorescent *in situ* hybridization study revealed the presence of monocentric centromeres across the soybean genome (Findley *et al.* 2010). Thus, the observation of more monocentric centromeres in the Hwangkeum assembly is evidence that despite the shorter total length of centromeres, the Hwangkeum assembly has been improved relative to the Williams 82 reference assembly in terms of overall scaffold order and position in the pericentromeric regions of the assembly.

### Phylogenetic analysis of centromeric satellite repeats

**Figure 3:**
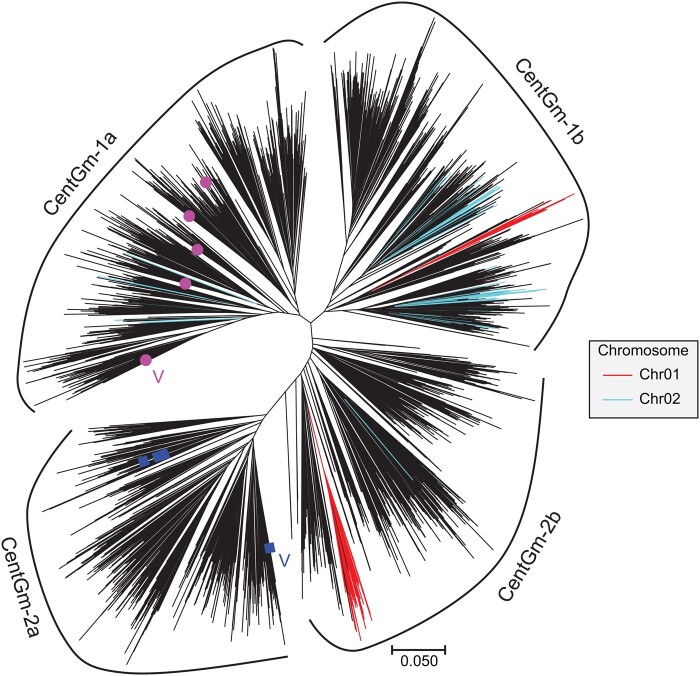
Neighbor-joining phylogenetic tree of 4469 centromeric repeat sequences in the Hwangkeum assembly together with nine publicly available representative sequences. Repeat sequences hit by BLAST searches with CentGm-2 or CentGm-1 were combined and then clustered with a cutoff of 90% similarity. Repeat clusters with lengths ranging from 88 to 95 bp were used for further analysis. Representative repeat sequences publicly available are indicated by pink circles for CentGm-1 and by blue squares for CentGm-1. The sequences used for BLAST searches were also highlighted by V. Centromeric repeat sequences were grouped into four subfamilies; CentGm-1a, CentGm-1b, CentGm-2a, and CentGm-1a. Sequences on chromosome 1 are indicated by red branches and those on chromosome 2 by light blue branches.

The distributions patterns of satellite repeats identified by the BLAST searches suggested potentially novel subfamilies of satellite repeats. To clarify relationships among the potentially novel subfamilies and the two known subfamilies, CentGm-1 and CentGm-2, we conducted a phylogenetic analysis of satellite repeats identified in the Hwangkeum assembly. For the phylogenetic analysis, repeat sequences <89 or >96 bp were removed from the combined set of 25,030 repeat sequences from the Hwangkeum assembly for the sake of alignment. Of the resultant 20,386 repeat sequences, 4469 nonredundant centromeric repeat sequences were used to construct a Neighbor-joining distance tree. Four major clusters were found (Figure 3), in contrast to the previous report that there were two major subfamilies of centromeric repeats in the soybean genome (Gill *et al.* 2009; Valliyodan *et al.* 2019). Because the representative repeat sequences previously reported belong to the two most distant subfamilies, CentGm-1 subfamily was renamed as CentGm-1a, and CentGm-2 as CentGm-2a. Of the two novel subfamilies between CentGm-1a and CentGm-2a, the subfamily next to CentGm-1a was referred to as CentGm-1b, and the subfamily next to CentGm-2a as CentGm-2b. An alignment of their extracted consensus sequences is presented in Supplementary Figure S4. The finding of the two novel subfamilies in this study was likely due to the fact that we used less stringent BLAST cut-off criteria with blast-short and gap penalty options, in addition to the cutoff of 60% sequence identity and 80% match length used in the previous studies. Interestingly, the observation of four repeat subfamilies are somewhat consistent with the hypothesis that the differential distributions of soybean satellite repeats may reflect the allopolyploid nature of soybean (Gill *et al.* 2009).

Major portions of repeat sequences in each of the chromosomes appeared to belong to two adjacent subfamilies, with exceptions of chromosomes 4 and 17 where the repeat sequences were spread over four subfamilies and three subfamilies, respectively (Figure 3 and Supplementary Figure S5). Most of the chromosomes do not contain one or two of these four centromeric repeat subfamilies. Dispersion of each of the four repeat subfamilies on a number of chromosomes may represent relics of ancestral arrays rather than the mixing of chromosomes or assembly errors. This result indicates that rapid and dynamic changes in the centromeric DNA after the formation of the tetraploids may have occurred preferentially within each of the chromosomes rather than the intermixing of chromosomes. Thus, our result is somewhat consistent with significant genetic variation within centromeric satellites and asymmetrical distribution of centromere organization among the three subgenomes observed in hexaploid wheat (Lee *et al.* 2005), providing additional evidence for the integrity of the Hwangkeum assembly.

### Identification of centromeric satellite repeats in *Glycine latifolia*

The weakness or absence of hybridization with satellite repeats to genomic DNA within a genus suggested the rapid divergence of centromeric satellite repeats (Lee *et al.* 2005; Gill *et al.* 2009; Ta *et al.* 2021), and in the case of rice relatives, novel divergent satellite repeats with low or no sequence similarity with CentO were isolated from several relatives. As genome sequence of *G. latifolia* (Liu *et al.* 2018), a perennial relative of soybean, is available, we searched CentGm repeats in the *G. latifolia* genome. Interestingly, we extracted 3107 nonredundant repeat sequences using CentGm-1 and CentGm-2. The percent identity of those sequences with CentGm-1 and CentGm-2 ranged from 67% to 83%, consistent with the previous Southern hybridization results (Gill *et al.* 2009). Examination of sequence regions containing *G. latifolia* repeat using the Tandem Repeat Finder indicated that, unlike the appearance of similar numbers of the 91- or 92-bp monomers in soybean, most of the repeats are 91-bp monomer (Supplementary File S4). Of the repeats detected by the Tandem Repeat Finder, 73 of the 90-bp repeats and 2944 of the 91-bp repeats were members of the set of 3107 repeats identified by the BLAST searches, and 92-bp repeats were absent in the 3107 set.

The 3107 repeat sequences were combined with five CentGm-1a representative sequences and four CentGm-2a representative sequences, which were previously reported (Gill *et al.* 2009; Findley *et al.* 2010; Valliyodan *et al.* 2019), and 10 sequences from each of the CentGm-1b and CentGm-2b subfamilies in Figure 3. The resultant 3046 repeat sequences were aligned, and a Neighbor-joining distance tree was constructed (Supplementary Figure S6). Interestingly, the diverse types of soybean sequences were clustered into one large group interspersed with *G. latifolia* repeat sequences. Unlike the sequence divergence between the 91 and 92-bp repeat units in soybean, the 90-bp repeat sequences were also interspersed with 91-bp repeat sequences. The results indicated that although further investigation will be required because the *G. latifolia* assembly contained a much lower number of repeats than the Hwangkeum or Williams 82 assemblies, *G. latifolia* genome likely contains significantly divergent CentGm-type centromeric satellite repeats, reflecting the evolutionary distance between the two species. Nevertheless, observation of a unique repeat group in the *G. latifolia* assembly might provide an opportunity to further test the hypothesis that differential distributions of soybean satellite repeats may reflect the allopolyploid nature of soybean (Gill *et al.* 2009).

### Annotation of the Hwangkeum genome and gene content comparison with other publicly available soybean genomes

Repetitive sequences made up 50.2% of the Hwangkeum genome (Table 1 and Supplementary Table S7). Long terminal repeat (LTR) transposable elements were the most abundant elements (83.8% of repetitive content), including the Gypsy (56.7% of repetitive content) and Copia (26.3% of repetitive content) families. The portion of the repetitive sequences in the Hwangkeum genome appeared to be lower than the 58.7% of the Williams 82 genome and the average of 54.5% of the 26 soybean genomes assembled using PacBio sequencing data (Liu *et al.* 2020). Even if the satellite tandem repeats (∼1.0%) detected in the 26 soybean genomes are excluded, the Hwangkeum genome contained at least 3% (approximately 30 Mb) lower amount of repetitive sequences than the reported soybean genomes. In addition to the collapse of centromeric satellite repeats described above, this result suggests that the assembly collapse of repetitive sequences is likely a main cause of the shorter total lengths of the Hwangkeum assemblies relative to those of the Williams 82 and other soybean assemblies (Tørresen *et al.* 2019).

A total of 79,870 transcripts for 58,550 protein-coding genes were found, which numbers are comparable to 88,647 transcripts for 61,303 genes in the reference soybean genome Wm82.a2.v1 (86,256 transcripts for 52,872 genes in the updated Wm82v4 assembly). Assessment of the annotation completeness with two BUSCO databases, eukaryota odb10 and embryophyta odb10, indicated that the gene content was effectively captured in the PromethION assembly (Supplementary Table S8): BUSCO analysis against eukaryota odb10 and embryophyta odb10 demonstrated 247/255 (96.9%) and 1562/1614 (96.8%) of BUSCO genes from the assembly, respectively. Of the 79,870 transcripts, 76,823 (96.2%) were associated with EggNOG functional categories (Supplementary Table S9), 56,212 (70.4%) had an InterPro match, 56,682 (71.0%) had a PFAM match, and 40,345 (50.5%) were assigned a gene ontology (GO) term (Supplementary File S5). We annotated 327 NLR genes, the genes of agronomically important superfamily, in the Hwangkeum assembly using the Seqping pipeline, which number is much lower than the 477 in the Williams 82 Wm82.a2.v1 assembly. As TGFam-Finder was recently used to annotate 66 additional NLR genes from the Williams 82 Wm82.a2.v1 assembly (Kim *et al.* 2020), we re-annotated the NLR genes using TGFam-Finder in the Hwangkeum assembly. A total of 503 NLR genes were annotated using TGFam-Finder in the Hwangkeum assembly with 176 additionally predicted genes (Supplementary File S6), resulting in a similar number of annotated NLR genes between the Hwangkeum and Williams 82 assemblies.

A total of 26,433 orthologous groups were identified between the Hwangkeum and Williams 82 assemblies using OrthoMCL. The Hwangkeum and Williams 82 assemblies possessed 24,977 and 25,445 orthologous groups, respectively. Of them, 23,989 orthologous groups (90.7%) existed in common between the Hwangkeum and Williams 82 assemblies. With the same criteria, about 4.0% of the Hwangkeum genes (988) and about 5.7% of the Williams 82 genes (1456) were lineage-specific orthologous groups in the Hwangkeum and Williams 82 genome, respectively. The portions of lineage-specific genes, which are dispensable genes in terms of pan-genome, are somewhat lower than those of the recent soybean pan-genome analysis (Liu *et al.* 2020) that showed that dispensable gene families accounted for an average of 19.1% of the genes in individual accessions. Thus, this result indicates a close relationship between Hwangkeum and Williams 82.

Finally, to test the quality of the Hwangkeum assembly down to the nucleotide level in the euchromatic regions, we examined the presence of known polymorphisms at genetic loci associated with golden seed color and strong SMV resistance, which are two characteristics of Hwangkeum, and whose genes have recently been characterized (Chen *et al.* 2002; Yang *et al.* 2010; Jeong and Jeong 2014; Redekar *et al.* 2016). To characterize seed coat and flower colors, Yang *et al.* (2010) developed 28 markers from eight enzyme-encoding gene families and a transcription factor that had been characterized as regulating anthocyanin biosynthesis or were homologous to the genes characterized in other plants. Those markers were mapped in a Hwangkeum by IT182932 population. We confirmed that Hwangkeum polymorphic sequences of the 28 markers were present in the Hwangkeum assembly at the chromosomal locations predicted by both the genetic mapping as well as the Williams 82 assembly (Supplementary Table S10). Thus, the results provide evidence for the high quality of the Hwangkeum assembly.

**Figure 4:**
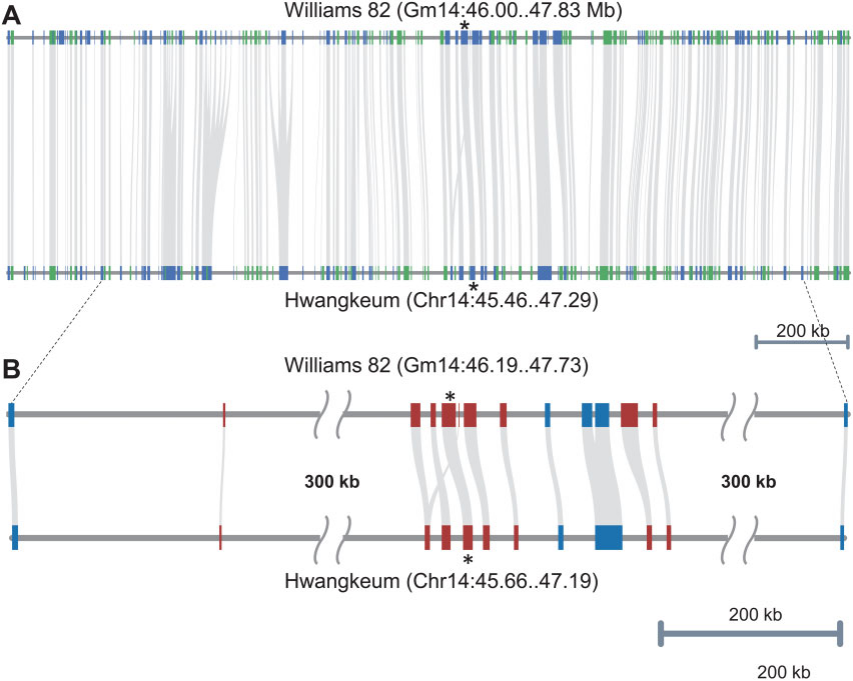
Comparison of gene arrangement between the Hwangkeum and Williams 82 assemblies at the chromosome 14 region in the vicinity of the *Rsv*3 locus. (A) Comparison of order and orientation of all homologous genes between the Williams 82 and Hwangkeum assemblies. Genes are indicated by blue and green boxes in an alternate manner. Homologs of the cloned *Rsv*3 genes are indicated by an asterisk. (B) Comparison of order and orientation of nucleotide-binding and leucine-rich-repeat (NLR) genes and leucine-rich repeat receptor-like kinase (LRR-RLK) genes that show a heterogeneous cluster. NLRs are indicated by red boxes and LRR-RLKs by blue boxes.

Hwangkeum is resistant to SMV, while Williams 82 is susceptible to SMV. The high level of resistance to all SMV strains in Hwangkeum was initially ascribed to a single dominant *Rsv*1 allele (Chen *et al.* 2002). However, Jeong and Jeong (2014) found that Hwangkeum contains more than two resistance genes at the classical *Rsv*1 locus as well as the *Rsv*3 locus. The two loci act in a complementary manner, in which the *Rsv*3 locus tends to confer resistance to SMV strains that are virulent to *Rsv*1-carrying plants. This locus is also interesting because it is located in the middle of a heterogeneous cluster (Suh *et al.* 2011) that contain members of the NLR as well as leucine-rich repeat receptor-like kinase (LRR-RLK) multigene families, of which some members have been reported to be disease resistance genes (Song *et al.* 1997; Parniske and Jones 1999). A strong candidate *Rsv*3 gene was proposed by a comparative sequence analysis (Redekar *et al.* 2016) and was then validated by overexpression and transient silencing (Tran *et al.* 2018; Ross *et al.* 2021). When the gene arrangement at this complex region spanning 1.83 Mb delimited by sequence-based markers Satt063 and GSINDEL133985 (Lee *et al.* 2013) was compared between the Hwangkeum and Williams 82 assemblies, the order and orientation of the shared genes were remarkably consistent with each other. Twenty-four of the 184 genes were unique to the Williams 82, and 12 of the 24 unique genes appeared to be functionally unannotated. In the case of the Hwangkeum assembly, 25 of the 168 genes were unique, and 23 of the 25 appeared to be functionally unannotated. Thus, those unique genes might have resulted from over-annotation of either assembly. The smaller total number of genes in the Hwangkeum is likely due to poor annotation in the multigene tandem repeat cluster by the Seqping pipeline because the TGFam-Finder added three more NLR genes at the *Rsv*3 locus. When the arrangement of only the NLR and LRR-RLK genes were examined between the two assemblies at this *Rsv*3 region, the order and orientation of the genes were consistent with each other, as we highlighted homologs of the cloned *Rsv*3 gene (Figure 4). The Williams 82 assembly contained one more partial NLR gene and one more LRR-RLK gene relative to the Hwangkeum. Interestingly, the Williams 82 Wm82.a2 version contained five LRR-RLK genes, while the Williams 82 Wm82.a1 version contained 10 LRR-RLK genes in our previous study (Suh *et al.* 2011), thereby indicating the much improved assembly in the Wm82.a2 version. Therefore, the high similarity of gene arrangement between the Hwangkeum and Williams 82 assemblies suggests that the gene-rich euchromatic regions of the Hwangkeum assembly are of a similar quality to those of the Williams 82 soybean reference genome sequence at the nucleotide level.

## Conclusions

In this study, we report the *de novo* assembly of the palaeopolyploid soybean genome through the integration of genetic linkage mapping and Nanopore PromethION sequencing. The total length of the present assembly (933 Mb) was shorter than that of the PacBio SMRT assembly of Hwangkeum (966 Mb) in this study as well as those of the public data (>970 Mb). The shorter assembly length is likely caused by assembly collapse at repeat regions (Tørresen *et al.* 2019), including centromeric satellite repeat regions as well as transposon repetitive sequences. However, several lines of evidence have suggested that the assembly quality of Hwangkeum at the chromosome level was more improved than the public assemblies. First, our enhanced detection of centromeric satellite repeats that resulted in a much greater number of repeats and the finding of two novel repeat subfamilies revealed more monocentric centromeres across all 20 chromosomes, which is consistent with the chromosomal nature of soybean genome predicted by the fluorescent *in situ* hybridization study (Findley *et al.* 2010), in the Hwangkeum assembly relative to the Williams 82 assembly. Second, we demonstrated that much shorter chromosomes or longer chromosomes could be explained by the predicted misjoints or insertions of unanchored scaffolds in the Williams 82 assembly, most of which were predicted by our previous genetic map study (Lee *et al.* 2020). Moreover, genetic markers or cloned genes associated with golden seed color and strong SMV resistance were located as predicted by previous genetic studies in the assembled chromosomes of Hwangkeum and the order and orientation of the examined genes were remarkably similar between the Hwangkeum and Williams 82 assemblies. Importantly, the BUSCO analyses indicated that the genome sequence and gene content qualities of our Hwangkeum assembly are comparable to those of the public assemblies. Thus, both the examinations of gene contents at genome-wide and specific chromosomal regions as an evolutionary measure of genome completeness suggest that the Hwangkeum assembly is a high-quality assembly. Different sequencing technologies show different pros and cons in the genome assembly projects (De Maio *et al.* 2019). Consequently, the present study shows that *de novo* genome assembly using the Nanopore PromethION long-reads platform provides promising results. Thus, this high-quality genome assembly for Hwangkeum will facilitate genetic dissection of the distinctive organoleptic and agronomical features of Hwangkeum, one of the typical cultivars in the Korean climate, as well as a better shaping of the soybean pan-genome.

## Data availability

All whole-genome sequencing data are available at NCBI (Bioproject PRJNA628825) except a previously reported set of paired-end short reads downloaded from NCBI with accession number: SRX6472178 (Kim *et al.* 2021). The genome assembly and annotation data of Hwangkeum v.1.0 is deposited at GenBank under the accession JAGRRG000000000. Supplementary material (Supplementary Figures S1–S5 and Supplementary Tables S1–S10) and six supplemental data Files are available at Figshare. The supplemental data Files are two Tandem Repeat Finder results (Supplementary Files S1 and S4), SNPs and indels (Supplementary File S2), SVs (Supplementary File S3), a list of annotated transcripts (Supplementary File S5), and a list of NLR genes (Supplementary File S6). Supplementary material is available at figshare: https://doi.org/10.25387/g3.14445140.

## Funding

This work was supported by the National Research Foundation grant (NRF-2018R1A2A2A05021904) funded by the Korea government awarded to S.-C.J. and by the Korea Research Institute of Bioscience and Biotechnology Research Initiative Program.

## Conflicts of interest

The authors declare that there is no conflict of interest.

## References

[jkab272-B1] Audano PA , Sulovari A, Graves-Lindsay TA, Cantsilieris S, Sorensen M, et al 2019. Characterizing the major structural variant alleles of the human genome. Cell. 176:663–675. 30661756 10.1016/j.cell.2018.12.019 PMC6438697

[jkab272-B2] Barra V , Fachinetti D. 2018. The dark side of centromeres: types, causes and consequences of structural abnormalities implicating centromeric DNA. Nat Commun. 9:4340. 30337534 10.1038/s41467-018-06545-y PMC6194107

[jkab272-B3] Bateman A , Martin MJ, O’Donovan C, Magrane M, Alpi E, et al 2017. UniProt: the universal protein knowledgebase. Nucleic Acids Res. 45:D158–D169. 27899622 10.1093/nar/gkw1099 PMC5210571

[jkab272-B4] Benson G. 1999. Tandem repeats finder: a program to analyze DNA sequences. Nucleic Acids Res. 27:573–580. 9862982 10.1093/nar/27.2.573 PMC148217

[jkab272-B5] Boetzer M , Henkel CV, Jansen HJ, Butler D, Pirovano W. 2011. Scaffolding pre-assembled contigs using SSPACE. Bioinformatics. 27:578–579. 21149342 10.1093/bioinformatics/btq683

[jkab272-B6] Camacho C , Coulouris G, Avagyan V, Ma N, Papadopoulos J, et al 2009. BLAST+: architecture and applications. BMC Bioinformatics. 10:421. 20003500 10.1186/1471-2105-10-421 PMC2803857

[jkab272-B7] Chakraborty M , Vankuren NW, Zhao R, Zhang X, Kalsow S, et al 2018. Hidden genetic variation shapes the structure of functional elements in Drosophila. Nat Genet. 50:20–25. 29255259 10.1038/s41588-017-0010-y PMC5742068

[jkab272-B8] Chan KL , Rosli R, Tatarinova TV, Hogan M, Firdaus-Raih M, et al 2017. Seqping: gene prediction pipeline for plant genomes using self-training gene models and transcriptomic data. BMC Bioinformatics. 18:1426. 28466793 10.1186/s12859-016-1426-6 PMC5333190

[jkab272-B9] Chan PP , Lowe TM. 2019. tRNAscan-SE: searching for tRNA genes in genomic sequences. Methods Mol Biol. 1962:1–14. 31020551 10.1007/978-1-4939-9173-0_1 PMC6768409

[jkab272-B10] Chen P , Buss GR, Tolin SA, Gunduz I, Cicek M. 2002. A valuable gene in Suweon 97 soybean for resistance to soybean mosaic virus. Crop Sci. 42:333–337.

[jkab272-B11] Chin CS , Peluso P, Sedlazeck FJ, Nattestad M, Concepcion GT, et al 2016. Phased diploid genome assembly with single-molecule real-time sequencing. Nat Methods. 13:1050–1054. 27749838 10.1038/nmeth.4035 PMC5503144

[jkab272-B12] Cuacos M , Franklin FCH, Heckmann S. 2015. Atypical centromeres in plants—What they can tell us. Front Plant Sci. 6:913. 26579160 10.3389/fpls.2015.00913 PMC4620154

[jkab272-B13] De Maio N , Shaw LP, Hubbard A, George S, Sanderson ND, et al 2019. Comparison of long-read sequencing technologies in the hybrid assembly of complex bacterial genomes. Microb Genom. 5:e000294. 10.1099/mgen.0.000294 PMC6807382 31483244

[jkab272-B14] Du J , Grant D, Tian Z, Nelson RT, Zhu L, et al 2010. SoyTEdb: a comprehensive database of transposable elements in the soybean genome. BMC Genomics. 11:113. 20163715 10.1186/1471-2164-11-113 PMC2830986

[jkab272-B15] English AC , Salerno WJ, Reid JG. 2014. PBHoney: Identifying genomic variants via long-read discordance and interrupted mapping. BMC Bioinformatics. 15:180. 24915764 10.1186/1471-2105-15-180 PMC4082283

[jkab272-B16] Findley SD , Cannon S, Varala K, Du J, Ma J, et al 2010. A fluorescence *in situ* hybridization system for karyotyping soybean. Genetics. 185:727–744. 20421607 10.1534/genetics.109.113753 PMC2907198

[jkab272-B17] Finn RD , Attwood TK, Babbitt PC, Bateman A, Bork P, et al 2017. InterPro in 2017-beyond protein family and domain annotations. Nucleic Acids Res. 45:D190–D199. 27899635 10.1093/nar/gkw1107 PMC5210578

[jkab272-B18] Fu L , Niu B, Zhu Z, Wu S, Li W. 2012. CD-HIT: accelerated for clustering the next-generation sequencing data. Bioinformatics. 28:3150–3152. 23060610 10.1093/bioinformatics/bts565 PMC3516142

[jkab272-B19] Geib SM , Hall B, Derego T, Bremer FT, Cannoles K, et al 2018. Genome annotation generator: a simple tool for generating and correcting WGS annotation tables for NCBI submission. Gigascience. 7:1–5. 10.1093/gigascience/giy018 PMC5887294 29635297

[jkab272-B20] Gill N , Findley S, Walling JG, Hans C, Ma J, et al 2009. Molecular and chromosomal evidence for allopolyploidy in soybean. Plant Physiol. 151:1167–1174. 19605552 10.1104/pp.109.137935 PMC2773056

[jkab272-B21] Holt C , Yandell M. 2011. MAKER2: an annotation pipeline and genome-database management tool for second-generation genome projects. BMC Bioinformatics. 12:491. 22192575 10.1186/1471-2105-12-491 PMC3280279

[jkab272-B22] Huerta-Cepas J , Szklarczyk D, Forslund K, Cook H, Heller D, et al 2016. EGGNOG 4.5: a hierarchical orthology framework with improved functional annotations for eukaryotic, prokaryotic and viral sequences. Nucleic Acids Res. 44:D286–D293. 26582926 10.1093/nar/gkv1248 PMC4702882

[jkab272-B23] Jeong N , Jeong S-C. 2014. Multiple genes confer resistance to soybean mosaic virus in the soybean cultivar Hwangkeum. Plant Genet Resour. 12:S41–S44.

[jkab272-B24] Jiang J , Birchler JA, Parrott WA, Dawe RK. 2003. A molecular view of plant centromeres. Trends Plant Sci. 8:570–575. 14659705 10.1016/j.tplants.2003.10.011

[jkab272-B25] Jiao WB , Schneeberger K. 2020. Chromosome-level assemblies of multiple *Arabidopsis* genomes reveal hotspots of rearrangements with altered evolutionary dynamics. Nat Commun. 11:989. 32080174 10.1038/s41467-020-14779-y PMC7033125

[jkab272-B26] Kim MS , Lozano R, Kim JH, Bae DN, Kim ST, et al 2021. The patterns of deleterious mutations during the domestication of soybean. Nat Commun. 12:97. 33397978 10.1038/s41467-020-20337-3 PMC7782591

[jkab272-B27] Kim S , Cheong K, Park J, Kim MS, Kim J, et al 2020. TGFam-Finder: a novel solution for target-gene family annotation in plants. New Phytol. 227:1568–1581. 32392385 10.1111/nph.16645 PMC7496378

[jkab272-B28] Kumar S , Stecher G, Tamura K. 2016. MEGA7: molecular evolutionary genetics analysis version 7.0 for bigger datasets. Mol Biol Evol. 33:1870–1874. 27004904 10.1093/molbev/msw054 PMC8210823

[jkab272-B29] Larkin MA , Blackshields G, Brown NP, Chenna R, Mcgettigan PA, et al 2007. Clustal W and Clustal X version 2.0. Bioinformatics. 23:2947–2948. 17846036 10.1093/bioinformatics/btm404

[jkab272-B30] Lee HR , Zhang W, Langdon T, Jin W, Yan H, et al 2005. Chromatin immunoprecipitation cloning reveals rapid evolutionary patterns of centromeric DNA in Oryza species. Proc Natl Acad Sci USA. 102:11793–11798. 16040802 10.1073/pnas.0503863102 PMC1187982

[jkab272-B31] Lee K , Kim M-S, Lee JS, Bae DN, Jeong N, et al 2020. Chromosomal features revealed by comparison of genetic maps of *Glycine max* and *Glycine soja*. Genomics. 112:1481–1489. 31461668 10.1016/j.ygeno.2019.08.019

[jkab272-B32] Lee WK , Kim N, Kim J, Moon J-K, Jeong N, et al 2013. Dynamic genetic features of chromosomes revealed by comparison of soybean genetic and sequence-based physical maps. Theor Appl Genet. 126:1103–1119. 23306355 10.1007/s00122-012-2039-8

[jkab272-B33] Lee YG , Jeong N, Kim JH, Lee K, Kim KH, et al 2015. Development, validation and genetic analysis of a large soybean SNP genotyping array. Plant J. 81:625–636. 25641104 10.1111/tpj.12755

[jkab272-B34] Li H. 2018. Minimap2: Pairwise alignment for nucleotide sequences. Bioinformatics. 34:3094–3100. 29750242 10.1093/bioinformatics/bty191 PMC6137996

[jkab272-B35] Li L , Stoeckert CJJ, Roos DS. 2003. OrthoMCL: Identification of ortholog groups for eukaryotic genomes. Genome Res. 13:2178–2189. 12952885 10.1101/gr.1224503 PMC403725

[jkab272-B36] Liu Q , Chang S, Hartman GL, Domier LL. 2018. Assembly and annotation of a draft genome sequence for *Glycine latifolia*, a perennial wild relative of soybean. Plant J. 95:71–85. 29671916 10.1111/tpj.13931

[jkab272-B37] Liu Y , Du H, Li P, Shen Y, Peng H, et al 2020. Pan-genome of wild and cultivated soybeans. Cell. 182:162–176. 32553274 10.1016/j.cell.2020.05.023

[jkab272-B38] Marçais G , Delcher AL, Phillippy AM, Coston R, Salzberg SL, et al 2018. MUMmer4: a fast and versatile genome alignment system. PLoS Comput Biol. 14:e1005944. 29373581 10.1371/journal.pcbi.1005944 PMC5802927

[jkab272-B39] Park KY , Hong EH, Chung KW, Hwang YH, Lee YH, et al 1981. New soybean variety “Hwangkeym-kong”. Agric Exp Stn Reports. 23:155–158.

[jkab272-B40] Parniske M , Jones JDG. 1999. Recombination between diverged clusters of the tomato Cf-9 plant disease resistance gene family. Proc Natl Acad Sci USA. 96:5850–5855. 10318973 10.1073/pnas.96.10.5850 PMC21949

[jkab272-B41] Redekar NR , Clevinger EM, Laskar MA, Biyashev RM, Ashfield T, et al 2016. Candidate gene sequence analyses toward identifying *Rsv*3-type resistance to soybean mosaic virus. Plant Genome. 9:10.3835/plantgenome2015.09.0088 27898808

[jkab272-B42] Ross BT , Flenniken ML, Ross BT. 2021. Extreme resistance to viruses in potato and soybean. Front Plant Sci. 12:658981. 33889169 10.3389/fpls.2021.658981 PMC8056081

[jkab272-B43] Ruan J , Li H. 2020. Fast and accurate long-read assembly with wtdbg2. Nat Methods. 17:155–158. 31819265 10.1038/s41592-019-0669-3 PMC7004874

[jkab272-B44] Saghai-Maroof MA , Soliman KM, Jorgensen RA, Allard RW. 1984. Ribosomal DNA spacer-length polymorphisms in barley: mendelian inheritance, chromosomal location, and population dynamics. Proc Natl Acad Sci USA. 81:8014–8018. 6096873 10.1073/pnas.81.24.8014 PMC392284

[jkab272-B45] Schmutz J , Cannon SB, Schlueter J, Ma J, Mitros T, et al 2010. Genome sequence of the palaeopolyploid soybean. Nature. 463:178–183. 20075913 10.1038/nature08670

[jkab272-B46] Shafin K , Pesout T, Lorig-Roach R, Haukness M, Olsen HE, et al 2020. Nanopore sequencing and the Shasta toolkit enable efficient *de novo* assembly of eleven human genomes. Nat Biotechnol. 38:1044–1053. 32686750 10.1038/s41587-020-0503-6 PMC7483855

[jkab272-B47] Shen Y , Du H, Liu Y, Ni L, Wang Z, et al 2019. Update soybean Zhonghuang 13 genome to a golden reference. Sci China Life Sci. 62:1257–1260. 31444683 10.1007/s11427-019-9822-2

[jkab272-B48] Shen Y , Liu J, Geng H, Zhang J, Liu Y, et al 2018. *De novo* assembly of a Chinese soybean genome. Sci China Life Sci. 61:871–884. 30062469 10.1007/s11427-018-9360-0

[jkab272-B49] Shimomura M , Kanamori H, Komatsu S, Namiki N, Mukai Y, et al 2015. The *Glycine max* cv. Enrei genome for improvement of Japanese soybean cultivars. Int J Genomics. 358127.2015:26199933 10.1155/2015/358127 PMC4493290

[jkab272-B50] Simão FA , Waterhouse RM, Ioannidis P, Kriventseva EV, Zdobnov EM. 2015. BUSCO: assessing genome assembly and annotation completeness with single-copy orthologs. Bioinformatics. 31:3210–3212. 26059717 10.1093/bioinformatics/btv351

[jkab272-B51] Song Q , Jenkins J, Jia G, Hyten DL, Pantalone V, et al 2016. Construction of high resolution genetic linkage maps to improve the soybean genome sequence assembly Glyma1.01. BMC Genomics. 17:33. 26739042 10.1186/s12864-015-2344-0 PMC4704267

[jkab272-B52] Song WY , Pi LY, Wang GL, Gardner J, Holsten T, et al 1997. Evolution of the rice Xa21 disease resistance gene family. Plant Cell. 9:1279–1287. 9286106 10.1105/tpc.9.8.1279 PMC156997

[jkab272-B53] Suh SJ , Bowman BC, Jeong N, Yang K, Kastl C, et al 2011. The rsv3 locus conferring resistance to soybean mosaic virus is associated with a cluster of coiled‐coil nucleotide‐binding leucine‐rich repeat genes. Plant Genome. 4:55–64.

[jkab272-B54] Swaminathan K , Varala K, Hudson ME. 2007. Global repeat discovery and estimation of genomic copy number in a large, complex genome using a high-throughput 454 sequence survey. BMC Genomics. 8:132. 17524145 10.1186/1471-2164-8-132 PMC1894642

[jkab272-B55] Ta TD , Waminal NE, Nguyen TH, Pellerin RJ, Kim HH. 2021. Comparative FISH analysis of Senna Tora tandem repeats revealed insights into the chromosome dynamics in Senna. Genes Genomics. 43:237–249. 33655486 10.1007/s13258-021-01051-w PMC7966213

[jkab272-B56] Tang H , Wang X, Bowers JE, Ming R, Alam M, et al 2008. Unraveling ancient hexaploidy through multiply-aligned angiosperm gene maps. Genome Res. 18:1944–1954. 18832442 10.1101/gr.080978.108 PMC2593578

[jkab272-B57] Tang H , Zhang X, Miao C, Zhang J, Ming R, et al 2015. ALLMAPS: robust scaffold ordering based on multiple maps. Genome Biol. 16:3. 25583564 10.1186/s13059-014-0573-1 PMC4305236

[jkab272-B58] Tek AL , Kashihara K, Murata M, Nagaki K. 2010. Functional centromeres in soybean include two distinct tandem repeats and a retrotransposon. Chromosome Res. 18:337–347. 20204495 10.1007/s10577-010-9119-x

[jkab272-B59] Tørresen OK , Star B, Mier P, Andrade-Navarro MA, Bateman A, et al 2019. Tandem repeats lead to sequence assembly errors and impose multi-level challenges for genome and protein databases. Nucleic Acids Res. 47:10994–11006. 31584084 10.1093/nar/gkz841 PMC6868369

[jkab272-B60] Tran PT , Widyasari K, Seo JK, Kim KH. 2018. Isolation and validation of a candidate Rsv3 gene from a soybean genotype that confers strain-specific resistance to soybean mosaic virus. Virology. 513:153–159. 29080441 10.1016/j.virol.2017.10.014

[jkab272-B61] van der Biezen EA , Jones JDG. 1998. The NB-ARC domain: a novel signalling motif shared by plant resistance gene products and regulators of cell death in animals. Curr Biol. 8:R226–R227. 9545207 10.1016/s0960-9822(98)70145-9

[jkab272-B62] Vahedian M , Shi L, Zhu T, Okimoto R, Danna K, et al 1995. Genomic organization and evolution of the soybean SB92 satellite sequence. Plant Mol Biol. 29:857–862. 8541510 10.1007/BF00041174

[jkab272-B63] Valliyodan B , Cannon SB, Bayer PE, Shu S, Brown AV, et al 2019. Construction and comparison of three reference-quality genome assemblies for soybean. Plant J. 100:1066–1082. 31433882 10.1111/tpj.14500

[jkab272-B64] Walker BJ , Abeel T, Shea T, Priest M, Abouelliel A, et al 2014. Pilon: an integrated tool for comprehensive microbial variant detection and genome assembly improvement. PLoS One. 9:e112963. 25409509 10.1371/journal.pone.0112963 PMC4237348

[jkab272-B65] Workman R , Fedak R, Kilburn D, Hao S, Liu K, et al 2018. High molecular weight DNA extraction from recalcitrant plant species for third generation sequencing. Protoc Exch. 1:1–15.

[jkab272-B66] Xie M , Chung CYL, Li MW, Wong FL, Wang X, et al 2019. A reference-grade wild soybean genome. Nat Commun. 10:1216. 30872580 10.1038/s41467-019-09142-9 PMC6418295

[jkab272-B67] Yang K , Jeong N, Moon JK, Lee YH, Lee SH, et al 2010. Genetic analysis of genes controlling natural variation of seed coat and flower colors in soybean. J Hered. 101:757–768. 20584753 10.1093/jhered/esq078

[jkab272-B68] Yang K , Moon J-K, Jeong N, Chun H-K, Kang S-T, et al 2011. Novel major quantitative trait loci regulating the content of isoflavone in soybean seeds. Genes Genom. 33:685–692.

[jkab272-B69] Yu YH , Yu H, Jeong J, Park H, Song D, et al 2008. A General Survey of Korean Legume Cultivars (in Korean). Suwon, Korea: National Institute of Crop Science.

